# The Role of miR-34a in the Hepatoprotective Effect of Hydrogen Sulfide on Ischemia/Reperfusion Injury in Young and Old Rats

**DOI:** 10.1371/journal.pone.0113305

**Published:** 2014-11-18

**Authors:** Xinli Huang, Yun Gao, Jianjie Qin, Sen Lu

**Affiliations:** Center of Liver Transplantation, The First Affiliated Hospital of Nanjing Medical University, The Key Laboratory of Living Donor Liver Transplantation, Ministry of Health, Nanjing, China; Virginia Commonwealth University, United States of America

## Abstract

Hydrogen sulfide (H_2_S) can protect the liver against ischemia-reperfusion (I/R) injury. However, it is unknown whether H_2_S plays a role in the protection of hepatic I/R injury in both young and old patients. This study compared the protective effects of H_2_S in a rat model (young and old animals) of I/R injury and the mechanism underlying its effects. Young and old rats were assessed following an injection of NaHS. NaHS alone reduced hepatic I/R injury in the young rats by activating the nuclear erythroid-related factor 2 (Nrf2) signaling pathway, but it had little effect on the old rats. NaHS pretreatment decreased miR-34a expression in the hepatocytes of the young rats with hepatic I/R. Overexpresion of miR-34a decreased Nrf-2 and its downstream target expression, impairing the hepatoprotective effect of H_2_S on the young rats. More importantly, downregulation of miR-34a expression increased Nrf-2 and the expression of its downstream targets, enhancing the effect of H_2_S on hepatic I/R injury in the old rats. This study reveals the different effects of H_2_S on hepatic I/R injury in young and old rats and sheds light on the involvement of H_2_S in miR-34a modulation of the Nrf-2 pathway.

## Introduction

Hepatic warm ischemia-reperfusion (I/R) injury is a dynamic process that frequently occurs during a variety of clinical situations, including liver transplantation and liver surgery [1]. A series of events, such as the formation of reactive oxygen species (ROS), depletion of ATP, production of inflammatory mediators, and apoptosis of hepatocytes are involved in the pathophysiology of hepatic I/R [2]. Several risk factors (aging and liver steatosis) can exacerbate liver failure during I/R [3]. Therefore, effective treatment of hepatic I/R injury is difficult.

The gasotransmitter hydrogen sulfide (H_2_S), similar to nitric oxide and carbon monoxide, is implicated in a wide range of physiological activities [4]. Endogenous H_2_S can be produced from L-cysteine in several organs, such as the brain, heart, kidney, and liver [5]. H_2_S has been reported to protect these tissues against I/R injury by maintaining mitochondrial function, inhibiting proinflammatory factors, neutralizing ROS and reducing apoptosis [6]. Treatment with H_2_S can be via inhalation of H_2_S or administration of NaHS by intravenous injection. However, it is difficult to control the concentration of inhaled H_2_S, resulting in potential toxicity to animals [7]. The administration of NaHS by intravenous injection has become the common treatment method in I/R injury because the concentration can be controlled [8].

MicroRNAs (miRNAs) are 20–25 nucleotides long non-coding RNAs that modulate a variety of biological processes, such as development, apoptosis, metabolism, and proliferation [9]. Aberrant miRNA expression is associated with a large number of pathophysiological conditions, including liver diseases [10]. In recent years, the role of miR-34a in the regulation of liver function and survival has received a great deal of attention [11]–[12]. An increase in the expression of miR-34a was reported to be involved in age-dependent loss of oxidative defense in the liver [13]. In addition, miR-34a expression was regulated in a partial hepatectomy model, which resulted in the inhibition of hepatocyte proliferation [14]. However, the role of miR-34a in hepatic I/R damage remains largely unknown.

As a target gene of miR-34a, nuclear erythroid-related factor 2 (Nrf-2) is involved in the detoxification process. Studies have shown that this transcription factor exerts an antioxidant effect by regulating the expression of antioxidant enzymes genes, such as glutathione S-transferase (GST), superoxide dismutases (SODs) and heme oxygenase-1 (HO-1), and NAD(P)H: quinine oxidoreductase-1 (NQO1) [15]–[17]. Nrf-2 provides cytoprotection by inducing an anti-inflammatory response [18]. It is activated by a variety of factors, including oxidative stress. The activation of Nrf-2 predominantly occurs via the release of the Nrf-2/Keap1 (Kelch-like ECH associating protein 1) complex in the cytosol of cells [19]. It has been reported that the administration of H_2_S can have beneficial effects on cardiac I/R injury by activating Nrf-2 [20]. In the liver, activation of Nrf-2 was reported to prevent or ameliorate toxin-induced injury and fibrosis [21].

Due to decreased endogenous antioxidants production and an increased inflammatory response, the ability of the liver cells of aged animals to combat I/R injury is significantly weakened [22]. Increasing evidence has shown that hepatic I/R injury is enhanced in aged animals and patients [23]. Although the effect of age on I/R-induced hepatic damage is well known, it is unknown whether the effect of therapy differs in aged and young patients with I/R injury. Here, we investigated if H_2_S protected the liver against I/R damage both in aged and young rats and whether miR-34a was involved in this effect.

## Materials and Methods

### Ethics statement

All the animals were treated humanely, using approved procedures in accordance with the guidelines of the Institutional Animal Care and Use Committee at Nanjing Medical University. The study was approved by the Experimental Animal Ethics Committee of Nanjing Medical University and the animal protocol was approved by the Ethics Review of Lab Animal Use Application of Nanjing Medical University (Permit Number: NJMU-ERLAUA-20120107).

### Chemicals and reagents

RPMI 1640 and DMEM were obtained from GIBCO (Invitrogen Company). Fetal bovine serum (FBS) was obtained from Hyclone (Logan, UT, USA). Lipofectamine 2000 transfection reagent was obtained from Invitrogen Life Technologies (Grand Island, NY, USA). NaHS was purchased from Sigma Aldrich (St. Louis, MO, USA). Antibodies against Nrf-2, GST, SOD, HO-1, and β-Actin were purchased from Santa Cruz Biotechnology (Santa Cruz, CA, USA). The Detergent Compatible (DC) Protein Assay kit was purchased from Bio-Rad Laboratories (Hercules, CA, USA).

### Animals and surgery

Young and old male Sprague-Dawley (SD) rats aged 3 months and 20 months, respectively, were obtained from Vital River Laboratories (VRL) in China. The young rats (3 months) were randomly and equally divided into six groups: sham, hepatic I/R, hepatic I/R +20 µmol/kg of NaHS, hepatic I/R+ a negative control oligonucleotide (NC), hepatic I/R+ an miR-34a mimic, hepatic I/R+NaHS+NC, and hepatic I/R+NaHS+an miR-34a mimic. The old rats (20 months) were also randomly and equally divided into six groups: sham, hepatic I/R, hepatic I/R +20 µmol/kg of NaHS, hepatic I/R+ anti-NC, hepatic I/R+ an miR-34a inhibitor, hepatic I/R+NaHS+anti-NC, and hepatic I/R+NaHS+an miR-34a inhibitor. The rats in the sham group underwent laparotomy, and the abdominal cavity was closed without hepatic I/R. Hepatic warm I/R was induced according to the previous reports [24]. Briefly, the rats were subjected to overnight fasting (with free access to water) before the surgery, and they were anaesthetized with 7% chloral hydrate (1 ml per 100 g of 7% chloral hydrate, intraperitoneal injection). After the midline laparotomy and anatomy of the hepatic portal, the left and median hepatic artery and the portal vein branches were blocked by no-damage artery clips to create a model of partial ischemia (70%). The right hepatic artery was opened to prevent the mesenteric venous congestion by permitting portal decompression through the right and caudate lobes. Under such circumstances of occluding, liver lobes were subjected to warm ischemia for 90 min. Reperfusion for 120 min was initiated by the removal of the clamp. The rats in the hepatic I/R+NaHS group received an intraperitoneal injection of 1 ml of NaHS solution 30 min before hepatic I/R. An miR-34a mimic (5′-UGGCAGUGUCUUAGCUGGUUGU-3′, 10 nmol) or miR-34a inhibitor (5′-ACAACCAGCUAAGACACUGCCA-3′, 10 nmol) in 0.1 ml of saline buffer was injected into the tail vein of the rats for 48 h before the administration of NaHS and subsequent liver I/R. A cholesterol-conjugated miR-34a mimic or an miR-34a inhibitor (both from RiboBio, Guangzhou, China) was used for in vivo RNA delivery. At each of the indicated time points (1, 3, 6 and 24 hours after I/R), six rats (per group) were randomly sacrificed, and blood and liver samples were collected.

### Measurement of serum H_2_S concentrations

Serum H_2_S concentrations were measured according to a previously reported method [24]. Briefly, 75 µl of sera were mixed with 300 µl of 10% trichloroacetic acid, 100 µL of distilled water and 150 µl of 1% zinc acetate. Then, 133 µl of N-dimethyl-p-phenylenediamine sulfate (20 µmol/L) and 133 µl of FeCl_3_ (30 µmol/L) were added to the mixture. After incubation at room temperature (25°C) for 15 min, the absorbance of the resulting solution was read at 670 nm. All the samples were assayed in duplicate, and serum H_2_S concentrations were calculated based on the calibration curve of NaHS.

### Measurement of serum ALT and AST

The serum samples were separated from rat blood by centrifugation at 1500 g for 15 min, and aspartate aminotransferase (AST) and alanine aminotransferase (ALT) were measured using an automated biochemistry analyzer (HITACHI 7600-020, Tokyo, Japan) to assess the hepatic function.

### Histopathological evaluation

Liver samples were frozen first and fixed in 10% neutral buffered formalin, embedded in paraffin, sliced into 5 µm thickness, and stained with hematoxylin-eosin. The histopathological scoring analysis was performed blindly according to previously described methods [25].

### Isolation of rat hepatocytes

Hepatocytes were isolated from the young and old rats according to a previous report [26]. Briefly, the liver was perfused retrogradely with 250 ml of 135 mmol/l NaCl, 7 mmol/l KCl, 12 mmol/l glucose, and 10 mmol/l HEPES, pH 7.4, followed by 250 ml of the same medium supplemented with collagenase (150 U/ml) and 1 mmol/l CaCl _2_. The Hepatocytes were diluted into William’s E medium supplemented with 10% fetal calf serum, 50 mg/ml penicillin-streptomycin, 5 mg/ml insulin and 4 ng/ml dexamethasone, 10 mmol/l HEPES and 1 mmol/l CaCl _2_.

### Western blot analysis

The rat hepatocytes and liver tissue samples were lysed with ice-cold lysis buffer containing the following: 50 mmol/l Tris-HCl, pH 7.4; 1% NP-40; 150 mmol/l NaCl; 1 mmol/l EDTA; 1 mmol/l phenylmethylsulfonyl fluoride; and complete proteinase inhibitor mixture (one tablet per 10 ml; Roche Molecular Biochemicals, Indianapolis, IN, USA). The lysates were sonicated using the Sonicator VCX130 (Sonics & Materials) on ice, followed by centrifuging at 12000 g for 10 minutes at 4°C and the supernatants were retained. The protein concentration in the cell lysate was quantified using the DC protein assay kit (Bio-Rad). After determination of the protein content with the DC Protein Assay kit. Western blot analysis was performed.

### Real-time PCR Assay

Mature miRNAs were isolated and purified using Trizol reagent (Invitrogen, USA), according to the manufacturer’s protocol. The levels of miRNAs (miR-34a, miR-28, miR-155, miR-27a, miR-144 and miR-153) were quantified with a TaqMan PCR kit. Real-time PCR was performed with LightCycler 480, using U6 small nuclear RNA as an internal normalized reference. The mature miRNAs were amplified using specific miR primers and an miScript universal primer (Qiagen, Hilden, Germany). The average expression levels of the miRNAs were normalized against U6 using the 2^−ΔΔCt^ method. Differences between the groups were presented as ΔCt, indicating the difference between the Ct value of the miRNAs and the Ct value of U6. To ensure consistent measurements throughout all the assays, for each PCR amplification reaction, three independent RNA samples were loaded as internal controls to account for any plate-to-plate variation, and the results from each plate were normalized against internal normalization controls.

The mRNA expression of Nrf-2, HO-1, NQO1, SOD2 and GST was assessed using SYBR GREEN PCR Master Mix (Applied Biosystems). The specific primers were as follows: Nrf-2 5′-GCTATTTTCCATTCCCGAGTTAC-3′ (forward), 5′-ATTG CTGTCCATCTCTGTCAG-3′ (reverse); HO-1 5′-CTTTCAGAAGGGTCAGGTG TC-3′ (forward), 5′-TGCTTGTTTCGCTCTATCTCC-3′ (reverse); NQO1 5′-CATCATTTGGGCAAGTCC-3′ (forward), 5′-ACAGCCGTGGCAGAACTA-3′ (reverse); SOD2 5′-GAGAAGTACCAGGAGGCGTTG-3′ (forward), 5′-GAGCCTTGGACACCAACAGAT-3′ (reverse); GST,5′-GCTCTATGGGAAG GACCAG-3′ (forward), 5′-CTCAAAAGGCTTCAGTTGC-3′ (reverse); GAPDH 5′-TATCGGACGCCTGGTTAC-3′ (forward), 5′-CTGTGCCGTTGAACTTGC-3′ (reverse). All the data were analyzed using GAPDH gene expression as an internal standard.

### Statistical analysis

The statistical analysis was performed with the statistical analysis software SPSS 13.0. Statistical analyses were performed using either an analysis of variance (ANOVA) or a Student’s *t*-test. Data were expressed as the mean ± standard deviation. *P*<0.05 was considered significant.

## Results

### H_2_S reduced hepatic I/R injury in young rats but has no effect in old rats

**Figure 1:**
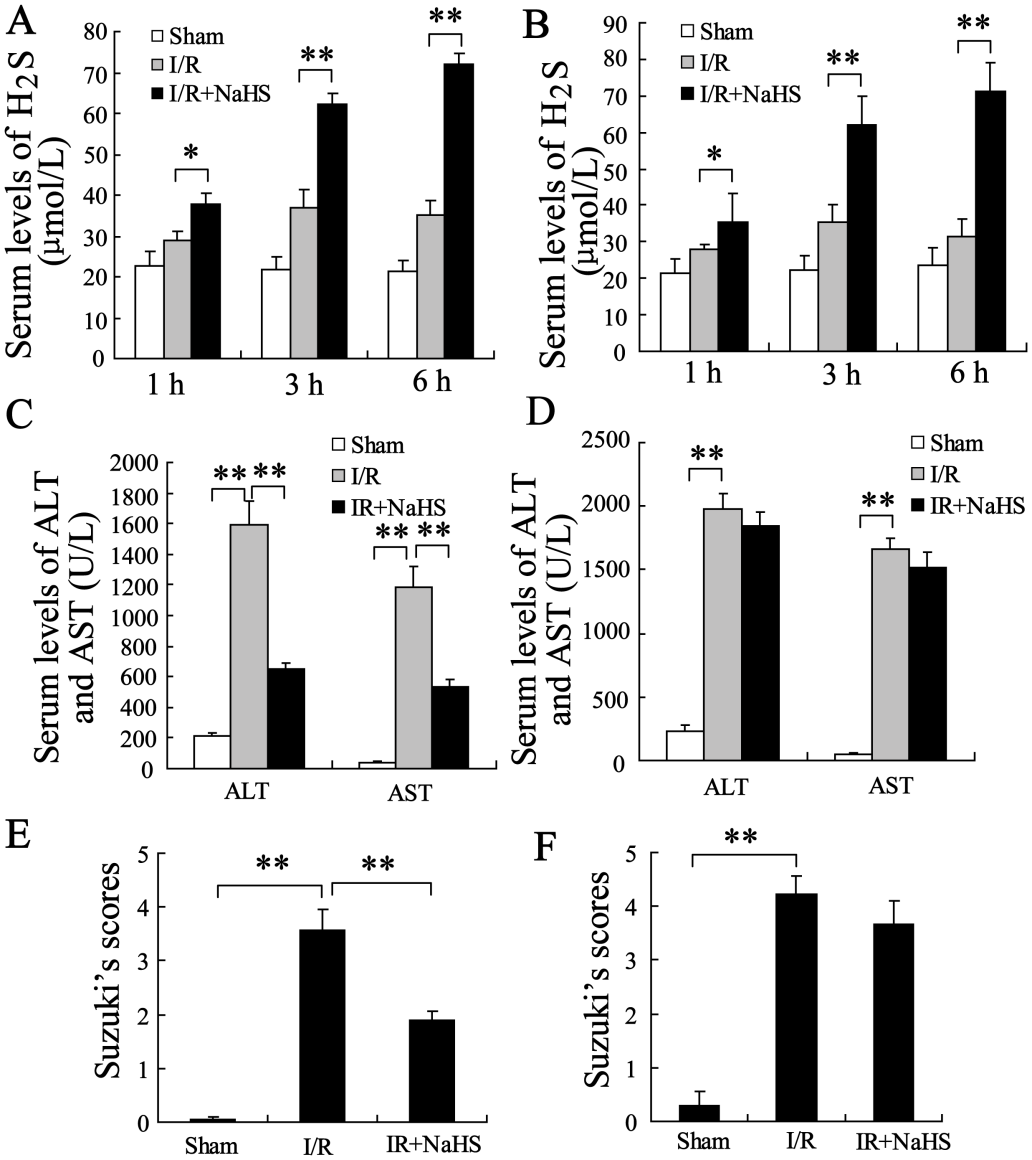
The effect of H_2_S on hepatic I/R injury in young and old rats. (A) The serum levels of H2S were significantly increased in the young rats that received a preconditioning dose of 20 µmol/kg NaHS compared to rats in the I/R group. (B) The serum levels of H2S were significantly increased in the old rats that received a preconditioning dose of 20 µmol/kg NaHS compared to the rats in the I/R group. (C) The serum levels for alanine aminotransferase (ALT) and aspartate aminotransferase (AST) were determined in the young rats after 6 h of reperfusion. (D) The serum levels for ALT and AST were determined in the old rats after 6 h of reperfusion. (E) Suzuki’s scores for the livers of the young rats after 24 h of reperfusion. (F) Suzuki’s scores for the livers of the old rats after 24 h of reperfusion. **P<0.01, indicates significant differences from the respective control groups.

To identify the effect of H_2_S on hepatic injury according to the age of the rats, the animals were treated with 20 µmol/kg NaHS. The serum levels of H_2_S, ALT and AST were measured 1, 3, and 6 hours after I/R. Treatment with NaHS 30 minutes prior to the ischemia markedly increased the serum concentration of H_2_S both in young and in old rats (Figure 1A and B, P<0.01). NaHS significantly reduced the serum levels of ALT and AST in young rats after 6 h reperfusion (Figure 1C, P<0.01). However, NaHS only slightly decreased the serum levels of ALT and AST in old rats (Figure 1D, P>0.05). These results imply that the protective effect of H_2_S on the hepatic I/R-induced damage differs in young and old rats. The level of ALT or AST in old rats was significantly higher than that of young rats without NaHS treatment, suggesting that old rats were prone to damage after hepatic I/R.

Hematoxylin and Eosin (HE) staining was performed on the liver tissues after 24 h of reperfusion. As shown in figure 1E and F, NaHS could improve liver damage in young rats (P<0.01) but had little effect in old rats (P>0.05).

### H_2_S stimulated Nrf-2-mediated signaling pathway in the hepatocytes of the young rats but has no effect in old rats

**Figure 2:**
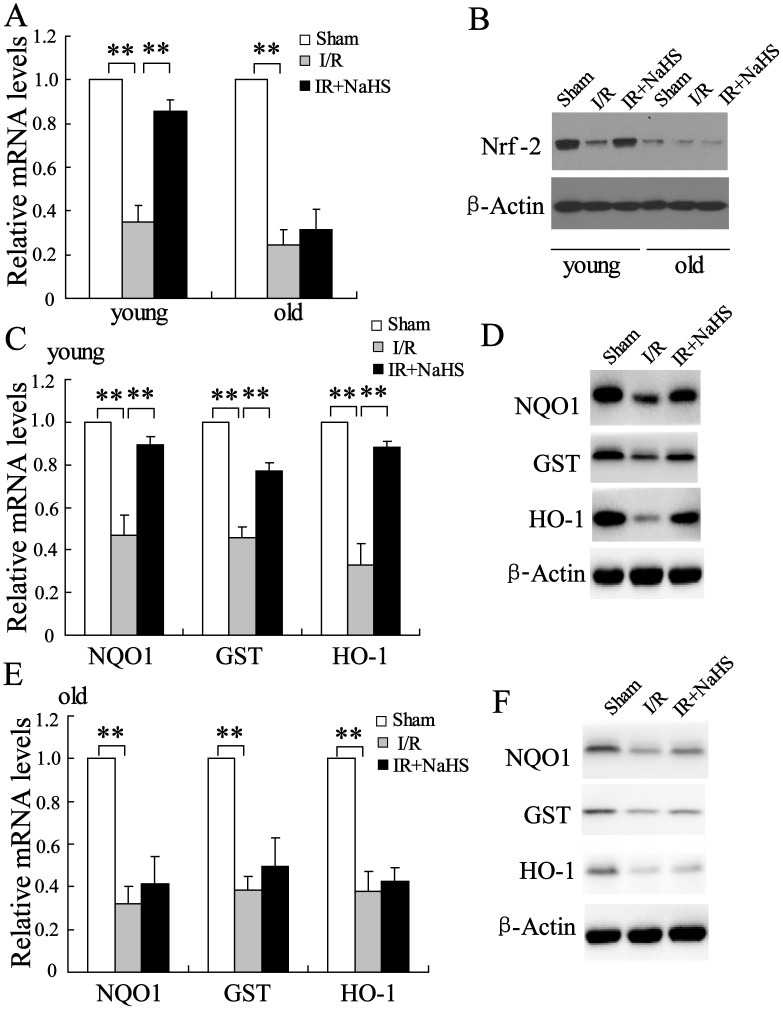
The effect of H_2_S on Nrf-2-mediated signaling pathway. Relative mRNA levels of Nrf-2 were assayed in the young and old rats. Pretreatment with NaHS (20 µmol/kg) significantly increased Nrf-2 mRNA (A) and protein (B) levels in the young rats treated with I/R, but it had little effect on those in the old rats. Pretreatment with 20 µmol/kg NaHS significantly increased mRNA (C) and protein (D) levels of NQO1, GST, and HO-1 in the young rats treated with I/R. Pretreatment of 20 µmol/kg NaHS slightly increased mRNA (E) and protein (F) levels of NQO1, GST and HO-1 in the old rats treated with I/R. **P<0.01, indicate significant differences from the respective control groups.

The antioxidant effects of the transcription factor Nrf-2 play a crucial role in the protection of hepatic I/R damage [27]. To measure whether Nrf-2 was involved in the effect of H_2_S on hepatic I/R, we measured the expression of Nrf-2 in the hepatocytes of young and old rats. The results showed that the mRNA level of Nrf-2 was significantly decreased in the hepatocytes of the young rats after 6 h I/R, and this decrease was reversed by NaHS treatment (P<0.01). The mRNA level of Nrf-2 was slightly changed in the hepatocytes of the old rats following I/R and the NaHS treatment compared to that of the untreated animals (Figure 2A, P>0.05). Consistent with the observed alteration in mRNA levels, NaHS treatment increased Nrf-2 protein levels in the hepatocytes of the young rats after I/R (Figure 2B). More importantly, the protein level of Nrf-2 in the hepatocytes of the young rats was significantly higher than that from old rats at baseline (Figure 2B).

It has been well documented that the transcription factor Nrf-2 up-regulates the expression of NQO1, GST and HO-1 [17]. Thus, we measured mRNA and protein levels of NQO1, GST and HO-1. After I/R, the expression of NQO1, GST and HO-1 was significantly reduced in the livers of the young rats, and the reduction was reversed by NaHS treatment (Figure 2C and 2D, P<0.01). However, no differences were observed in mRNA and protein levels of NQO1, GST and HO-1 in the old rats (Figure 2E and 2F, P>0.05). These results indicate that H_2_S can stimulate Nrf-2-mediated signaling pathway to protect the liver against I/R injury in young rats.

### H_2_S reduced miR-34a expression in hepatocytes of the young rats but has no effect in old rats

**Figure 3:**
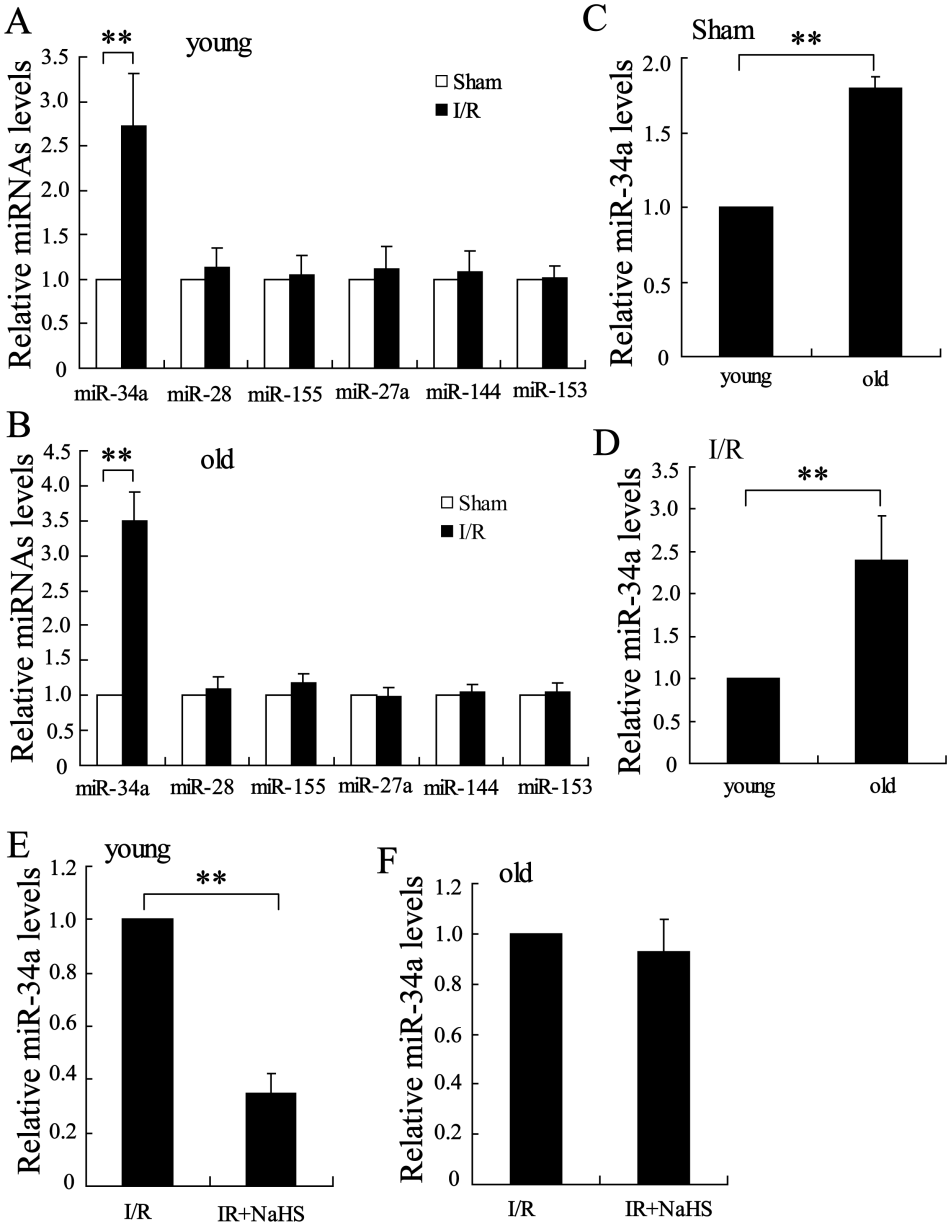
The effect of H_2_S on miR-34a expression in the I/R liver. Relative levels of miR-34a, miR-28, miR-155, miR-27a, miR-144, and miR-153 were assayed in young rats (A) and old rats (B) after I/R. Levels of miR-34a were lower in the young rats in the sham group (C) and the in I/R group (D) than in the old rats. (E and F) Pretreatment with 20 µmol/kg NaHS significantly decreased miR-34a levels in the young rats (E), but it had little effect on those in the old rats. **P<0.01, indicates significant differences from the respective control groups.

To further study the mechanism of H_2_S on Nrf-2 expression in the liver after I/R, we detected the expression of many miRNAs including miR-34a, miR-28, miR-155, miR-27a, miR-144 and miR-153, which may be involved in regulating the expression of this transcription factor [28]. Real-time PCR assays showed that, among these miRNAs, miR-34a was significantly up-regulated in the hepatocytes of the young and old rats after I/R (Figure 3A and B). Interestingly, the level of miR-34a was remarkably increased in the old rats compared to that in the young rats in the sham group and the I/R group (Figure 3C and D, P<0.01).

We also measured the expression of miR-34a in the liver following I/R and treatment with NaHS. As shown in Figure 3E and 3F, NaHS significantly decrease miR-34a expression in hepatocytes from young rats after I/R, but it had no effect on miR-34a expression in the hepatocytes of the old rats.

### Overexpression of miR-34a could inhibit hepatoprotective effect of H_2_S on young rats

**Figure 4:**
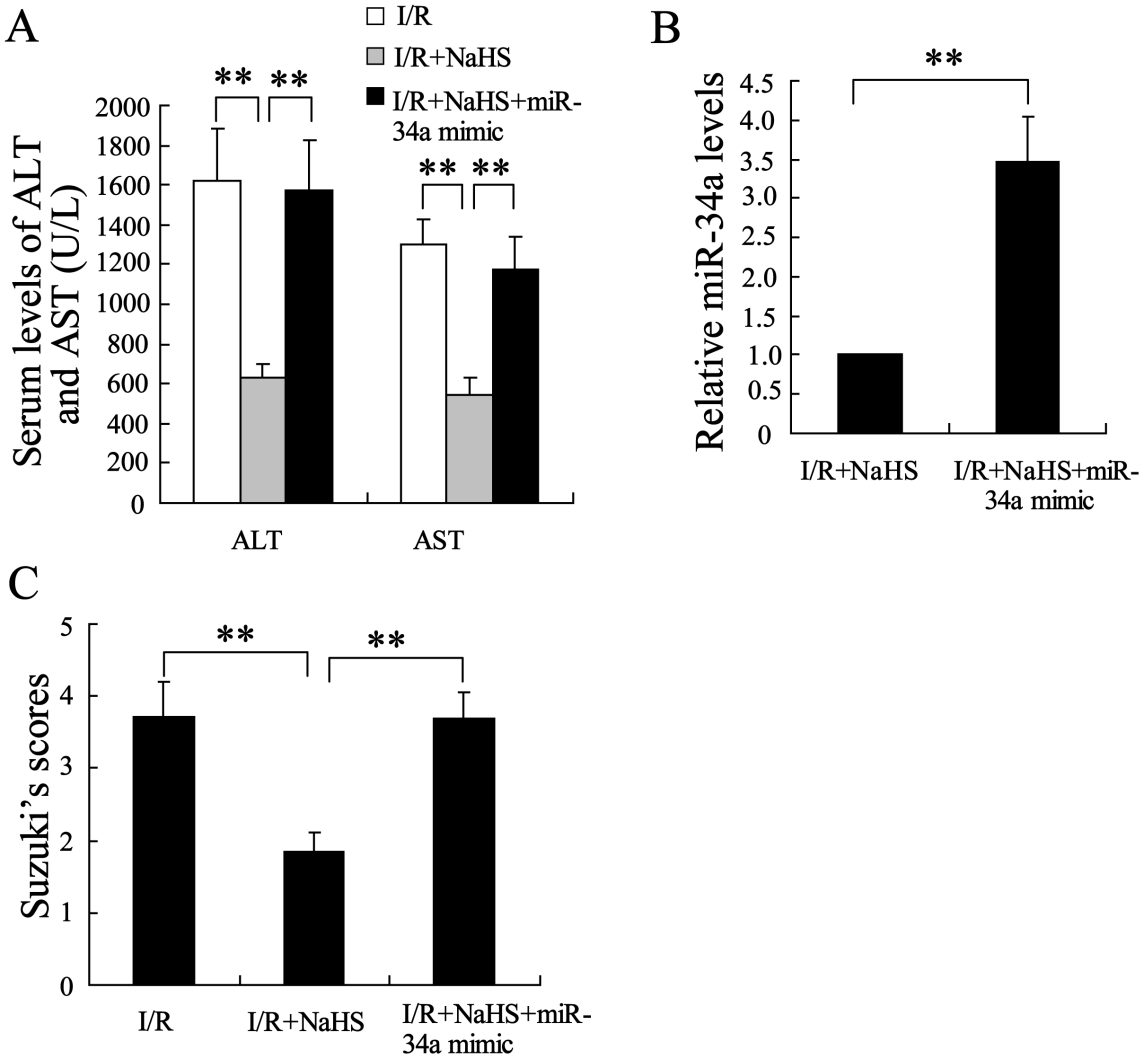
Overexpression of miR-34a inhibited the hepatoprotective effect of H_2_S on young rats. (A) The serum levels of ALT and AST were assayed in the young rats. The serum levels of ALT and AST were significantly decreased in the young rats following the pretreatment with NaHS, and this decrease was reversed by miR-34a mimic. (B) The injection of miR-34a mimic clearly increased miR-34a levels. (C) Suzuki’s scores for the livers of the young rats. **P<0.01, indicates significant differences from the respective control groups.

Next, the role of miR-34a in the hepatoprotective effect of H_2_S on young rats was further explored. After a tail vein injection of the cholesterol-conjugated miR-34a mimic, a slight increase in the serum levels of ALT and AST was observed in hepatic I/R young rats. However, the miR-34a mimic significantly reversed the effect of H_2_S on hepatic I/R injury (Figure 4A, P<0.01). In addition, the expression of miR-34a was significantly increased in the hepatocytes of the young rats administered NaHS and the miR-34a mimic (Figure 4B, P<0.01). These results indicate that miR-34a was involved in the hepatoprotective effect of H_2_S on hepatic I/R in the young rats.

### Knockout of miR-34a expression enhanced the hepatoprotective effect of H_2_S on old rats

**Figure 5:**
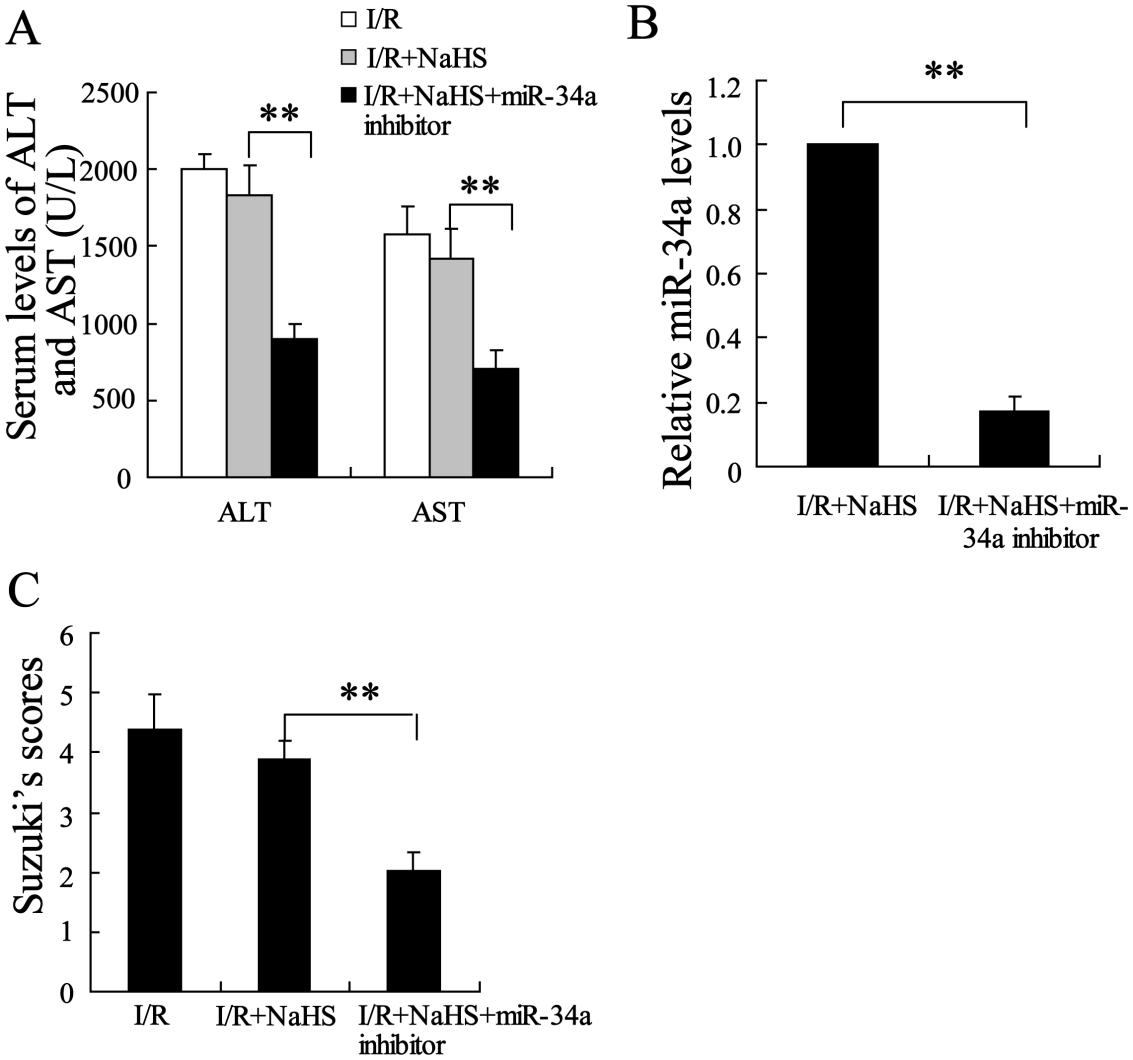
Knockout of miR-34a expression enhanced the hepatoprotective effect of H_2_S on the old rats. (A) The serum levels of ALT and AST were assayed in the old rats. Pretreatment with NaHS had little effect on the serum levels of ALT and AST, which were significantly decreased by miR-34a inhibitor in the old rats with I/R. (B) The injection of miR-34a inhibitor clearly decreased miR-34a levels. (C) Suzuki’s scores for the livers of the old rats. **P<0.01, indicates significant differences from the respective control groups.

As the expression of miR-34a is abundant in hepatocytes of the old rats, we wondered whether the hepatoprotective effect of H_2_S would be enhanced if its expression was down-regulated by an miR-34a inhibitor. As expected, the administration of the miR-34a inhibitor administration decreased serum levels of ALT and AST in the old rats with hepatic I/R. Hepatoprotective effect were improved in the I/R group treated with NaHS and the miR-34a inhibitor compared to those in the miR-34a inhibitor-only group (Figure 5A, P<0.01), with the inhibitor further attenuating the pathological changes in the livers of the NaHS-treated old rats (Figure 5B, P<0.01).

### miR-34a mediation of Nrf-2 signaling pathway was implicated in the hepatoprotective effects of H_2_S

**Figure 6:**
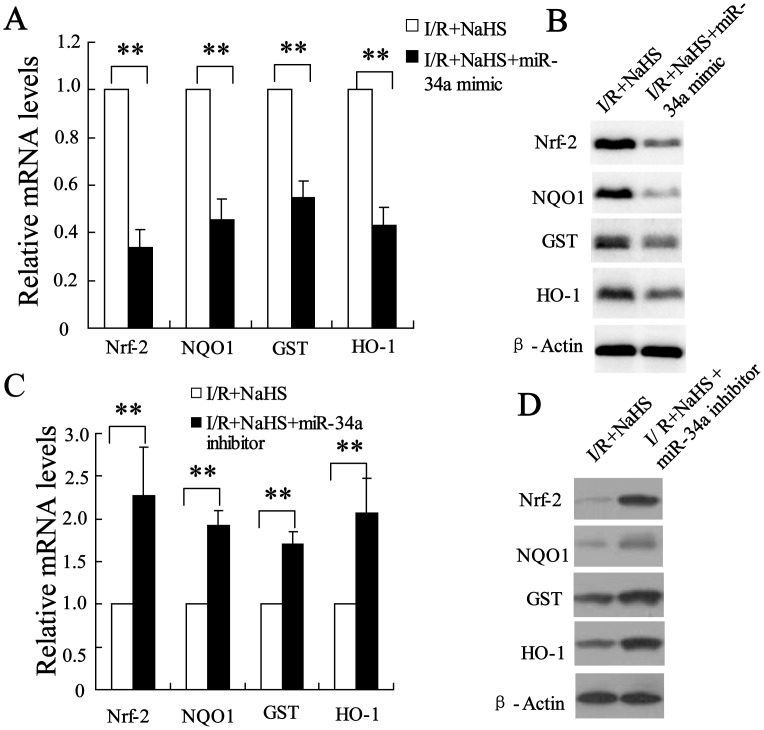
miR-34a mediated Nrf-2 signaling pathway was implicated in the hepatoprotective effects of H_2_S. The injection of miR-34a mimic decreased the mRNA (A) and protein (B) levels of Nrf-2, NQO1, GST and HO-1 in the young rats in the I/R+pretreatment with NaHS group. The injection of miR-34a inhibitor increased the mRNA (C) and protein (D) levels of Nrf-2, NQO1, GST and HO-1 in the old rats in the with I/R+pretreatment with NaHS group. **P<0.01, indicates significant differences from the respective control groups.

To explore the mechanism of miR-34a in the hepatoprotective effects of H_2_S, the expression of Nrf-2, NQO1, GST and HO-1 was measured in the young and old rats after injection of miR-34a mimic or inhibitor. As shown in Figure 6A and 6B, miR-34a mimic could reduce the expressions of Nrf-2, NQO1, GST and HO-1 in the young rats after hepatic I/R pretreated with NaHS, suggesting that the promotion of Nrf-2-mediated signaling pathway by NaHS might act through down-regulation of the miR-34a level. On the other hand, miR-34a inhibitor significantly increased the expressions of Nrf-2, NQO1, GST and HO-1 in the old rats after hepatic I/R injury with NaHS treatment (Figure 6C and 6D), suggesting that miR-34a-mediated Nrf-2 signaling pathway is involved in hepatoprotective effects of H_2_S.

## Discussion

The gasotransmitter H_2_S can protect several tissues including the liver against I/R injury [29]. Previous study focused on the protective effect of H_2_S on the tissues of young animals. The present study explored the hepatoprotective effect of H_2_S on young (3 months) and old rats (20 months). We found that NaHS alone could reduce hepatic I/R injury in young rats, but it had little effect on hepatic I/R injury in old rats. In addition, NaHS pretreatment decreased miR-34a expression in the hepatocytes of the young rats treated with hepatic I/R. Our data also showed that miR-34a was implicated in H_2_S-induced prevention of liver damage in the young rats. More importantly, the inhibition of miR-34a expression enhanced the effect of H_2_S on hepatic I/R injury in the old rats. H_2_S might promote Nrf-2-mediated signaling pathway through the down-regulation of the expression of miR-34a. The levels of miR-34a were higher in the hepatocytes of the old rats than in those of the young rats.

Due to its anti-inflammatory, antioxidative, and cytoprotective activity, H_2_S is capable of protecting tissues from I/R-induced injury [30]. The present study has for the first time explored the effect of H_2_S on hepatic I/R injury in young and old rats. Our data showed that the administration of NaHS, a donor of H_2_S, significantly decreased serum levels of AST and ALT, as well as histopathological alterations after hepatic I/R in young rats. However, NaHS had little effect on I/R-induced liver injury in the old rats. The different effect of NaHS on I/R injury was due to decreased production of endogenous antioxidants with increasing age. Our data demonstrated that NaHS stimulated the expression of Nrf-2 and its downstream target gene in young rats but that it had little effect on the expression of these molecules in old rats.

MiR-34a was previously reported to be involved in the regulation of liver function and survival [11]. In this study, we found that the level of miR-34a was remarkably higher in the hepatocytes of the old rats compared to that of the young rats, which was consistent with previous findings [12]. NaHS significantly decreased miR-34a expression in the hepatocytes of the young rats but had little effect on miR-34a expression in the old rats due to the hepatoprotective effect of H_2_S. To investigate the relationship between miR-34a and the effect of NaHS on hepatic I/R, we used miR-34a mimic and miR-34a inhibitor. Injection with miR-34a mimic diminished the protective effect of NaHS on hepatic I/R injury in the young rats. In the old rats, the combination of NaHS and the miR-34a inhibitor prevented the damage caused by hepatic I/R. MiR-34a has been implicated in liver oxidative stress during aging [12]. Based on that, we believe that the oxidative stress defense function of NaHS might rely on the regulation of miR-34a expression.

Our data also indicated that miR-34a mediation of the Nrf-2 signaling pathway was implicated in the hepatoprotective effect of H_2_S. There are several lines of evidence to support this. First, in the young rats, miR-34a mimic reduced the expression of Nrf-2 and its downstream target gene in hepatic I/R pretreated with NaHS. Second, miR-34a inhibitor significantly increased the expression of Nrf-2 and its downstream target gene in the old rats with or without NaHS pretreatment. Third, miR-34a level was negatively correlated with Nrf-2 expression, which is consistent with the finding of a previous report [13].

Our data demonstrated that I/R stress decreased HO-1 mRNA and protein expressions in rat liver (Figure 2), which is inconsistent with the previous report [31]. It is possible that chloral hydrate treatment instead of pentobarbital treatment might affect the expression of some antioxidant gene in the liver. However, more clarification of the specific molecular mechanism involved is needed.

In summary, we demonstrated that NaHS had a different effect on hepatic I/R damage in young and old rats. Our results also suggested that the hepatoprotective effect of NaHS in the young rats was due to decreased miR-34a expression, which resulted in the promotion of Nrf-2 signaling pathway. The protective effect of NaHS when it was combined with miR-34a inhibitor in the old rats provided further evidence for the role of miR-34a in hepatic I/R. These results may lead to the development of therapeutic strategies to minimize injury after I/R during liver transplantation and liver surgery both in young and aged patients.
